# The effect of anxiety and depression on the health-related quality of life of severe acute pancreatitis survivors: structural equation modeling approach

**DOI:** 10.3389/fpsyt.2023.1160807

**Published:** 2023-05-02

**Authors:** Xueting Wang, Weili Zhan, Ling Huang, Yunmei Guo, Yousha Wang, Huiwen Tan, Lianhong Wang

**Affiliations:** ^1^Nursing Department, Affiliated Hospital of Zunyi Medical University, Zunyi, China; ^2^Department of Critical Medicine, Affiliated Hospital of Zunyi Medical University, Zunyi, China; ^3^Nursing College, Zunyi Medical University, Zunyi, China

**Keywords:** severe acute pancreatitis, quality of life, anxiety, depression, structural equation modeling

## Abstract

**Background:**

Understanding the relationship between anxiety, depression and health-related quality of life (HRQOL) provides important clues to alleviate anxiety, depression and improve HRQOL in patients after severe acute pancreatitis (SAP). The aim of this study was to examine the effects of anxiety and depression on HRQOL in post-SAP patients using structural equation modeling.

**Methods:**

A cross-sectional study design was used and 134 patients with SAP from the Affiliated Hospital of Zunyi Medical University were recruited. Data collected included demographic and clinical characteristics, the English Standard Short Form 36 (SF-36) Health Survey, The Self-rating Anxiety Scale (SAS) and The Self-rating Depression Scale (SDS). Structural equation modeling analysis was conducted using the AMOS 24.0 program.

**Results:**

The mean of HRQOL score was 49.42 (SD = 23.01). The prevalence of anxiety and depression in post-SAP patients was 33.6 and 34.3%, respectively. Both anxiety and depression have a direct negative impact on HRQOL (β = −0.360, *p* < 0.001; β = −0.202, *p* = 0.034). Anxiety also negatively affects HRQOL indirectly through depression (β = −0.118, *p* = 0.043). The analysis of the covariance structure revealed that the resulting model had a reasonable goodness of fit.

**Conclusion:**

Anxiety and depression reduce the quality of life of SAP patients during recovery. Regular assessment and management of the anxiety and depression status of SAP patients is necessary and will help them improve their HRQOL more effectively.

## Introduction

Severe acute pancreatitis (SAP) is the most serious subtype of acute pancreatitis (AP) and associated with persistent organ failure (more than 48 h) and systemic inflammatory response syndrome. The global pooled incidence of acute pancreatitis is 34 cases per 100,000 of the general population per year (1), of which, 20–30% will develop into SAP (2). The majority of patients with SAP have at least 2 weeks of ICU experience with 25% mortality (3). Although medical advances have contributed to a significant decrease in mortality from SAP, survivors still face many problems. Following SAP, approximately one-third of patients developed dyspepsia due to pancreatic exocrine insufficiency (4), 30% developed diabetes within 6 years of follow-up (5), 24% of patients had persistent pain for 1 year (6), and 21% of patients relapsed after the first acute pancreatitis episode (1). They also face the threat of changes in family life, financial worry and fewer job opportunities (7). This set of issues underscores the long-term nature of the disease, with survivors of SAP being prone to health-related quality of life (HRQOL) decline and mental disorders.

It is generally considered that HRQOL reflects the impact of disease, treatment and patient perceptions on daily functioning and the individual’s ability to conduct a fulfilling life (8). The range of questions identified through HRQOL contributes to improving the treatment and care of patients (9). Studies have shown that lower HRQOL is always associated with a poor prognosis and that improving the quality of life is effective in reducing complication rates and readmission rates in SAP patients (10). It is essential to consider HRQOL in patients after SAP. However, former research has shown that survivors of SAP have lower quality of life scores than the general population 3 years (11, 12). Elder, female, poor digestion and significant weight loss were considered to have worse HRQOL (13).

Health-related quality of life of SAP patients is affected not only by the disease itself and demographic factors, but also by mental disorders. There is a high risk of mental disorders in SAP patients, of which anxiety and depression are very common (14, 15). A systematic review indicated that the prevalence of anxiety and depression in AP patients was 33.4 and 36.8%, respectively (16). Anxious patients often lose the confidence to resume their daily lives and constantly torn between hope and despair, which make them prone to a lack of recovery initiative (17). Affected by depression, patients lose interest in life and are easily pessimistic and tired. Studies have shown that anxiety and depression can lower SAP patients’ pain threshold, increase digestive reactions, reduce sleep quality, and lead to decreasing immune function (7, 18), which may diminish the HRQOL.

In fact, only a few studies have investigated mental health after SAP. A cross-sectional study of SAP in China found depression to be associated with low quality of life (19), but the causal relationship is unclear. Anxiety has not been introduced into HRQOL studies of SAP survivors, but numerous studies of other systemic disorders have demonstrated that anxiety is an influential factor of HRQOL. Anxiety is always associated with a poorer prognosis, and we believe that anxiety also affects HRQOL in SAP survivors, but the magnitude of its effect is unclear. In addition, we know that the occurrence of anxiety and depression are always highly correlated. Early Freud observed that when the ego is involved in a difficult mental task, energy control is compromised, energy expenditure is reduced in other ways, and a general state of inhibition or depression may occur. In severe cases, anxiety helps to produce inhibition in order to avoid the dangers associated with impulsive behavior (20). Subsequently, some studies suggest that anxiety may be a precursor to later depression (21, 22). Therefore, we suggest that anxiety can also affect HRQOL through depression, but the value of its indirect effects should be elucidated in further analyses.

A better understanding of their relationship is important to provide clues to relieve anxiety and depressive and improve HRQOL in patients after SAP, which may also decrease the risk for their adverse associated complications. This study aimed to use structural equation modeling (SEM) to examine the effect of anxiety and depression on the HRQOL in patients after SAP. SEM is an approach for building, estimating, and testing causal models (23). SEM clearly analyzes the role of individual indicators on the aggregate and the interrelationships among individual indicators, which is an appropriate method to examine the relationship among anxiety, depression and HRQOL in SAP survivors in this study. Based on previous research, we propose a initial model for SAP survivors in which (i) anxiety directly affects HRQOL; (ii) depression directly affects HRQOL; and (iii) anxiety indirectly affects HRQOL through depression.

## Materials and methods

### Study setting and population

We conducted a cross-sectional study of patients with SAP at the Affiliated Hospital of Zunyi Medical University. It is a general hospital with over 3,000 beds, providing healthcare to about 12 million people in the region. The hospital’s intensive care unit (ICU) is the treatment center for severe acute pancreatitis in Guizhou Province. We recruited patients with SAP who were treated in the ICU or hepatobiliary surgery between May 2021 and October 2022. Diagnosis was based on the Atlanta Classification System requiring (24). The criteria for inclusion included: (i) 18 years of age or older; (ii)successfully discharged from the hospital through treatment; and (iii)voluntary participation in this study and signed informed consent form. Patients with clear psychiatric (e.g., schizophrenia, depression, anxiety, etc.) and cognitive problems prior to the onset of SAP and those who refused to participate in the study were not included. This study was approved by the Ethics Committee of the Affiliated Hospital of Zunyi Medical University, China (KLL-2022-005).

### Measurements

Self-designed general information questionnaire includes demographic and clinical characteristics. Demographic characteristics included age, gender, height, weight, marital status, occupation, education level, current smoking and drinking status. Clinical characteristics including etiology, APACHE II score, opioid use, access to psychotherapy and common comorbidities such as hypertension, diabetes, hyperlipidemia, cardiovascular disease (stroke/heart disease/heart attack). After the questionnaires were collected, body mass index (BMI) was calculated by the researchers based on height and weight. Age and gender will be included in the model as control variables according to previous studies. Dyspepsia and weight loss as one of the items assessed for depression, we do not include control variables here.

The HRQOL of participants was evaluated using the English Standard Short Form 36 (SF-36) Health Survey. SF-36 Health Survey, the most commonly used tool to assess HRQOL in patients with pancreatitis (25). This scale is used by patients to assess their health status through recalling their life status in the last 4 weeks. There are 36 items on this scale, divided into 8 dimensions: physical functioning (PF), role-physical (RP), bodily pain (BP), general health (GH), vitality (VT), social functioning (SF), role-emotional (RE), and mental health (MH). The score for each dimension will be converted between 0 and 100, and the total HRQOL score will be the average of all dimension score. The higher score indicating better HRQOL. In this study, the Cronbach’s alpha coefficient for each dimension was 0.82–0.91.

The Self-rating Anxiety Scale (SAS) was utilized to assesses anxiety. This scale consists of 20 items with four-point Likert-type options, and participants were invited to select the option that matched their level of feeling. To convert the highest score into 100 points, the final score is 1.25 times the actual total score. The final score is 50–59 for mild anxiety, 60–69 for moderate anxiety, and 70 or more for severe anxiety. The Cronbach’s alpha coefficient for this scale was 0.86 in the present study.

Depression was measured using the Self-rating Depression Scale (SDS), which consists of 20 items with 10 positive and 10 negative questions. To convert the highest score into 100 points, the final score is 1.25 times the actual total score. Mild depression scored 50 to 59, moderate depression scored 60 to 69, and major depression scored 70 and above. In this study, the Cronbach’s alpha coefficient for this scale was 0.89.

### Data collection

All data was collected by reviewing the patient’s medical records and using a structured questionnaire. The structured questionnaire contains demographic characteristics, opioid use, psychological treatment status and the SF-36 Health Survey, SAS and SDS. Due to the limitations of regional epidemic management, most patients are advised to choose the nearest hospital for follow-up. The day before patients were discharged, we obtained their informed consent and added them or their carer as a “friend” contact on WeChat. WeChat is a social networking platform that allows users to connect with each other using text, photo sharing, voice and video calls and currently has over 1.2 billion monthly active users (26). We set up the questionnaire on the platform of the online survey tool Questionnaire Star.^1^ We obtained the link to the questionnaire and sent it to the participants via WeChat, where they could click on the link and then fill in the questionnaire online. For patients who could not use WeChat, we sought the help of their carers to assist them in filling out the questionnaire. Data collected by 2 trained researchers, and at the end of data collection, questionnaires were checked for completeness daily. To ensure the quality of questionnaire completion, a link to the questionnaire was sent to the patient’s or their carer’s WeChat the day before discharge to enable the patient to train once (the results of this questionnaire completion were not included in the analysis). The researcher accompanies the patient during this process and answers the patient’s questions without inducing the patient to make a choice, ensuring that the patient is able to complete the questionnaire correctly and alone after discharge.

### Statistics

The data were analyzed using SPSS18.0. In descriptive analysis, standard deviations (SD), frequency and percentages are used to view the distribution of the data. Before hypothesis testing, checking the internal consistency reliability of measurements using Cronbach’s alpha. Data normality was also examined by kurtosis and skewness. Pearson’s correlation coefficient was used to test for correlation and collinearity between the variables. AMOS 24.0 was used to construct SEM to examine the relationships among exogenous, endogenous, mediating, and control variables. The analysis was started with the hypothesized model, and modify it by adjusting the path. The path coefficients were calculated using standardized regression coefficients. The fit indices were used to assess the fit of the data to the hypothetical model, including Chi-square (χ^2^), normal Chi-square (χ^2^/df), Goodness of Fit Index (GFI), normed fit index (NFI), comparative fit index (CFI), root mean squared error of approximation (RMSEA), incremental fit index (IFI); and Akaike’s information criterion (AIC). The Bootstrapping method was used to test the statistical significance of the direct, indirect and total effects of the hypothetical model.

## Result

### Sample characteristics

There were 159 patients who agreed to participate in the study, and 134 patients completed the survey 1–12 months after SAP, with a response rate of 84.28%. Of the patients who withdrew, there were 3 deaths within 3 months of discharge (1 from cirrhosis and the others from pancreatitis), and 1 death from pancreatitis within 6 months of discharge, while 12 could not be contacted and 9 requested to withdraw from the study. Baseline characteristics of study participants are shown in Table 1. The average age of participants was 44 years (range: 20–72), and there were more males (57.46%) than females (42.54%) and about three out of four patients were overweight. About half of the participants had at least a high school education, and only a small percentage were unemployed. There were 89.55% of participants who were married. Of the participants, 69.4% of patients had their first episode of AP. Hyperlipidemia (36.57%) was the most common comorbidity among the participants, followed by hypertension (20.89%), diabetes (14.92%), and cardiovascular disease (5.97%), while 22.39% of the participants had two or more comorbidities. After SAP, only 2.24% of patients were current drinkers, 51.49% were current smokers, and no patients were using opioids or receiving psychotherapy.

**TABLE 1:** Baseline characteristics of study participants (n = 134).

Variables	Categories	*n*	%
Age (years)	<30	15	11.19
	30–60	107	79.85
	>60	12	8.96
Gender	Male	77	57.46
	Female	57	42.54
BMI	Low (<18.5)	3	2.25
	Normal (18.5∼22.9)	28	20.89
	Overweight (≥23)	103	76.86
Marital status	Married	120	89.55
	Unmarried/Divorce/Widowed	14	10.45
Occupation	Employee	57	42.54
	Freelancer	42	31.34
	Retirement	5	3.73
	Unemployed	30	22.39
Education level	Graduated junior high school and below	70	52.24
	Graduated high school	35	26.12
	Graduated university and above	29	21.64
Current smoker	Yes	69	51.49
	No	65	48.51
Current drinker	Yes	3	2.24
	No	131	97.76
Aetiology	Biliary	62	46.27
	Alcohol	17	12.67
	Hypertriglyceridemic	49	36.57
	Idiopathic	5	3.74
	Gestational	1	0.75
Comorbidities	Hypertension	28	20.89
	Diabetes	20	14.92
	Hyperlipidemia	49	36.57
	Cardiovascular disease	8	5.97
Prior AP	Yes	41	30.60
	No	93	69.40
APACHE II score	Mean ± SD	12.32 ± 2.45	

BMI, body mass index; APACHE II score, acute physiology and chronic health evaluation II score.

### HRQOL, anxiety, and depression

Participants had lower levels of HRQOL and higher incidence of anxiety and depression. The mean of HRQOL score was 49.42 (*SD* = 23.01). The average score of each dimension from highest to lowest is: mental health (60.06 ± 18.33), physical functioning (58.54 ± 26.18), bodily pain (56.06 ± 30.87), social functioning (52.13 ± 27.64), vitality (51.49 ± 19.77), role-emotional (47.26 ± 36.63), general health (40.16 ± 17.69), and role-physical (30.6 ± 29.82). For anxiety, the mean score was 46.94 (*SD* = 10.62). The prevalence of anxiety in patients after SAP was 33.6% (95% *CI*: 25.5%, 41.7%). Among them, the incidence of mild, moderate, and severe anxiety was 21.6, 6.0, and 6.0%, respectively. The mean score of depression was 46.98 (*SD* = 10.86). The prevalence of depression in SAP patients was 34.3% (95% *CI*: 26.2%, 42.5%). The incidence of mild, moderate, and severe depression was 23.1, 6.0, and 5.2%, respectively. Pearson’s correlation coefficient between the variables have been presented in Table 2. No correlation between control variables (age and gender) and other variables. Both anxiety and depression were negatively correlated with HRQOL, and there was a positive correlation between anxiety and depression. The maximum correlation matrix between the exogenous and endogenous variables was 0.584, with no multicollinearity problem.

**TABLE 2:** Means, standard deviations, and correlations between observed variables.

Variables	M	SD	HRQOL	Anxiety	Depression	Age	Gender
HRQOL	49.42	23.01	1				
Anxiety	46.94	10.62	-0.490**	1			
Depression	46.98	10.86	-0.459**	0.584**	1		
Age	44.39	12.14	0.86	-0.106	-0.41	1	
Gender	-	-	0.25	-0.100	-0.145		1

*N* = 134.

**p < 0.01.

### Analysis of the hypothetical model

The model include one exogenous variable (SAS), one mediator variable (SDS), two control variables (age, gender), and one endogenous variable (HRQOL). Initial testing of the hypothetical model resulted in a poor fit to the data: χ^2^ = 193.098, χ^2^/*df* = 3.786, GFI = 0.813, NFI = 0.842, CFI = 0.877, RMSEA = 0.145, IFI = 0.878, and AIC = 247.098. No significant effects of the control variables (age and gender) on the dependent variables were found. To optimize the model, we removed paths that were not statistically significant or had path normalized load values below 0.5 from. The testing of the modified model showed that all the paths were statistically significant (*P* < 0.05), The final model fit results are acceptable: χ^2^ = 24.549; χ^2^/*df* = 1.444, GFI = 0.958; NFI = 0.973; CFI = 0.992; RMSEA = 0.058; IFI = 0.992; AIC = 62.549 (Table 3).

**TABLE 3:** Bootstrap analysis of direct, indirect and total effects in AMOS.

Parameter	Estimate	SE	Ratio of total effect	95% CI	
				**LLCL**	**ULCL**
Standardized direct effect	−0.36**	0.096	75.31%	−0.554	−0.174
Standardized indirect effect	−0.118*	0.062	24.68%	−0.251	−0.003
Standardized total effect	−0.478**	0.068	–	−0.606	−0.341

SE, Standard error.

**: *p* < 0.01; *: *p* < 0.05.

### Path analysis for the model

**FIGURE 1:**
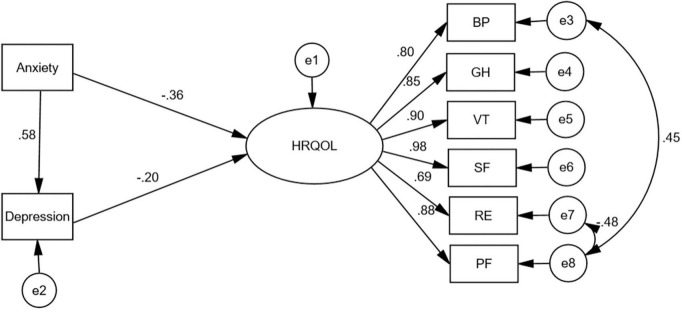
Structural equation model of health-related quality of life among SAP survivors.

Bootstrap analysis (*B* = 5,000) of direct, indirect and total effects in AMOS. Mediated effects analysis was conducted with anxiety as the independent variable, depression as the mediating variable, and HRQOL as the dependent variable (Table 3). The results indicated: anxiety not only directly predicted HRQOL, but also predicted HRQOL through the mediating effect of depressive effects, with the direct and mediating effects accounting for 75.31 and 24.68% of the total effect, respectively. There were significant direct effects of anxiety (β = −0.360, *p* < 0.001), depression (β = −0.202, *p* = 0.034) on HRQOL. Anxiety had significant indirect effect (β = −0.118, *p* = 0.043) on HRQOL. Anxiety has a direct effect on depression (β = 0.584, *p* < 0.001) (Figure 1).

## Discussion

This study proposed a hypothetical model of HRQOL in patients after SAP, and tested the model through SEM to identify the effect of anxiety and depression on HRQOL. Direct comparisons with the results of other studies are difficult because there are no other HRQOL models of SAP survivors. The results of this study show that the current status of HRQOL, anxiety and depression in patients after SAP are undesirable. Anxiety and depression substantially impact the HRQOL of patients.

Our study investigated the HRQOL status of patients with SAP within 1 year after hospital discharge. The mean score of HRQOL in SAP survivors was 49.42 ± 23.01, which was clearly lower than the Chinese norm (HRQOL:78.18 ± 15.88) (27), it shows that the physical and psychological problems faced during the recovery period have a great impact on the patients. Among all the dimensions, the role-physical was the worst. those results are approximately the same as those in Pittsburgh and Newcastle (11, 28). Compared with the previous study, the scores of the five dimensions of physical functioning, physical pain, vitality, social function, and mental health increased, and the scores of the three dimensions of role-physical, general health, and role-emotional remained the same as before. These findings suggest that HRQOL levels in SAP patients still need to be improved, especially to improve role functioning.

Our results showed that the prevalence of anxiety and depression in SAP patients was 33.6 and 34.3%, respectively. The prevalence of anxiety and depression in SAP patients was similar to that of AP patients and did not increase as the disease became more severe (16). Probably because they face the same risks of complications and life challenges (4, 29). Clinical studies have shown that people with anxiety and depression are more likely to have poor treatment adherence, unhealthy eating patterns, and irregular sleep habits (30, 31). Prolonged experience of these unhealthy lifestyles can exacerbate psychological problems, leading to lower quality of life and poor outcomes, trapped in a vicious cycle. The high incidence of anxiety and depression in SAP patients shows that it is important to pay attention to their psychological problems.

The final model proposed in this study had a sound impact on the data, suggesting that the model is an appropriate framework to explain the effects of anxiety and depression on HRQOL in patients after SAP. We found that age and gender had no impact on HRQOL, which is inconsistent with the result of previous study suggesting that elder and female predicted HRQOL limitation (13). This difference may be related to the fact that the age of the patients in this study was mainly distributed among middle and young adults. This may also indicate that there is now a trend toward a younger age of onset of SAP. Second, a national follow-up study of 1,068 AP patients found that men and women were equally likely to develop complications (32). This may explain the lack of difference in their quality of life. In addition, we found that anxiety and depression has a negative effect on HRQOL, Higher the level of anxiety and depression, lower the HRQOL.

The most influential psychological factor in predicting HRQOL in patients after SAP is anxiety. Anxiety has a direct and indirect effect on HRQOL. This result suggests that anxiety itself can affect the HRQOL of patients after SAP. In a survey of patients’ perceptions of their experience of recovery from acute pancreatitis, the stress of limited ability to perform daily living, the uncertainty of the disease, and frustration with the recovery process were found to be the main causes of anxiety. Anxiety is one of the main reasons for the decline in quality of life for most patients (7), supporting the results of this study. Anxiety may also indirectly affect HRQOL through depression, with depression playing a partially mediating role in both, this is consistent with the results of previous studies (21, 22). Research shows that anxious people may use avoidance as a coping strategy to reduce their negative emotions. Whereas avoidance may reduce the likelihood of experiencing positive events and activities, which may ultimately lead to depression (33). Furthermore, research has shown that anxiety decreases the effectiveness of pain treatment, which in turn is one of the main causes of depression (34). Sarkar et al. (35) showed that the incidence of depression in patients with pain was twice as high as in non-pain patients. Therefore, consideration of anxiety risk management may lead to more effective depression and HRQOL management measures.

Regarding the relationship between depression and HRQOL, results demonstrate that depression is an important factor for low HRQOL. This finding is in line with previous study (19). Pain, digestive symptoms are among the causes of depression (35). In addition, lack of knowledge about pancreatitis, uncertainty about the disease and fear of recurrence, life stress and lack of family support can all contribute to emotional exhaustion (36). These patients may present with fatigue, feelings of helplessness, lack of interest in things, guilt and shame and despair (37). Depression is known to be a major risk for suicide. A cross-sectional study in Taiwan found that patients with pancreatic disease suffering from depression had a higher suicidal mortality rate (42.89%) (18). Concomitant psychiatric disorders (e.g., depression) also predicted readmission in patients with pancreatitis (38). Thus, it is necessary for medical care providers to regularly assess patients’ mental status and prevent the onset of depression to promote the improvement of HRQOL.

Studies have shown that only 21% of patients with AP actively follow medical advice, and more patients require the active assistance of medical staff in the long term (35). In practice, there are a few long-term follow-up studies aimed at improving HRQOL in AP patients that focus on interventions for complications and do not include psychological interventions (39, 40). The detrimental effects of anxiety and depression on HRQOL in AP patients are often overlooked, which may provide a potential factor for the poor state of anxiety and depression and the compromised improvement of HRQOL. Long-term overall care is necessary, not just a focus on physical health, and this study provides a new perspective on the need to incorporate psychological care to promote HRQOL in post-SAP patients. There are several limitations of this study. Firstly, due to epidemic control, the data collected in this study were self-administered questionnaires based on WeChat or telephone, and about half of the patients included in the study had junior secondary education or less, which had limitations in ensuring the objectivity of the questionnaire data. When conditions permit, researchers will be encouraged to use face-to-face follow-up surveys in the future. Secondly, the study was conducted in a single center, which may limit the generalizability of the results. Therefore, for future studies we propose to conduct in multi-center. And finally, the cross-sectional study limited the breadth and depth of the research to a certain extent. The HRQOL and mental status of patients are dynamic, and future longitudinal studies on HRQOL and anxiety and depression in SAP survivors are recommended to understand their developmental trajectory.

## Conclusion

Both anxiety and depression have a direct negative impact on HRQOL. Anxiety also negatively affects HRQOL indirectly through depression. The higher the score of anxiety and depression, the lower the level of HRQOL. Regular assessment and management of anxiety and depression status in SAP patients is essential. Early intervention and treatment services with modifiable factors will more effectively help them to improve HRQOL.

## Data availability statement

The raw data supporting the conclusions of this article will be made available by the authors, without undue reservation.

## Ethics statement

The studies involving human participants were reviewed and approved by the Ethics Committee of the Affiliated Hospital of Zunyi Medical University. The patients/participants provided their written informed consent to participate in this study.

## Author contributions

LW, XW, and WZ: conception or design of the study. LH, YG, YW, and HT: acquisition, analysis, or interpretation of data for the work. XW and WZ: drafting the work or revising it critically for important intellectual content and agreement to take responsibility for all aspects of the work to ensure accuracy and completeness. LW: approval of the final version to be published. All authors contributed to the article and approved the submitted version.

## Footnotes

1
https://www.wjx.cn/
